# Supplementary material for: Effectiveness and Safety of Ayurvedic Medicines in Type 2 Diabetes Mellitus Management: A Systematic Review and Meta-Analysis
Source: Front Pharmacol. 2022 Jun 8;13:821810. doi: 10.3389/fphar.2022.821810 (PMC9213670; doi:10.3389/fphar.2022.821810)
Supplement: Supplementary file 1 [file DataSheet1.zip › Supplementary Material/Forest and Funnel Plots/Zingiber officinale/DBP.pdf]

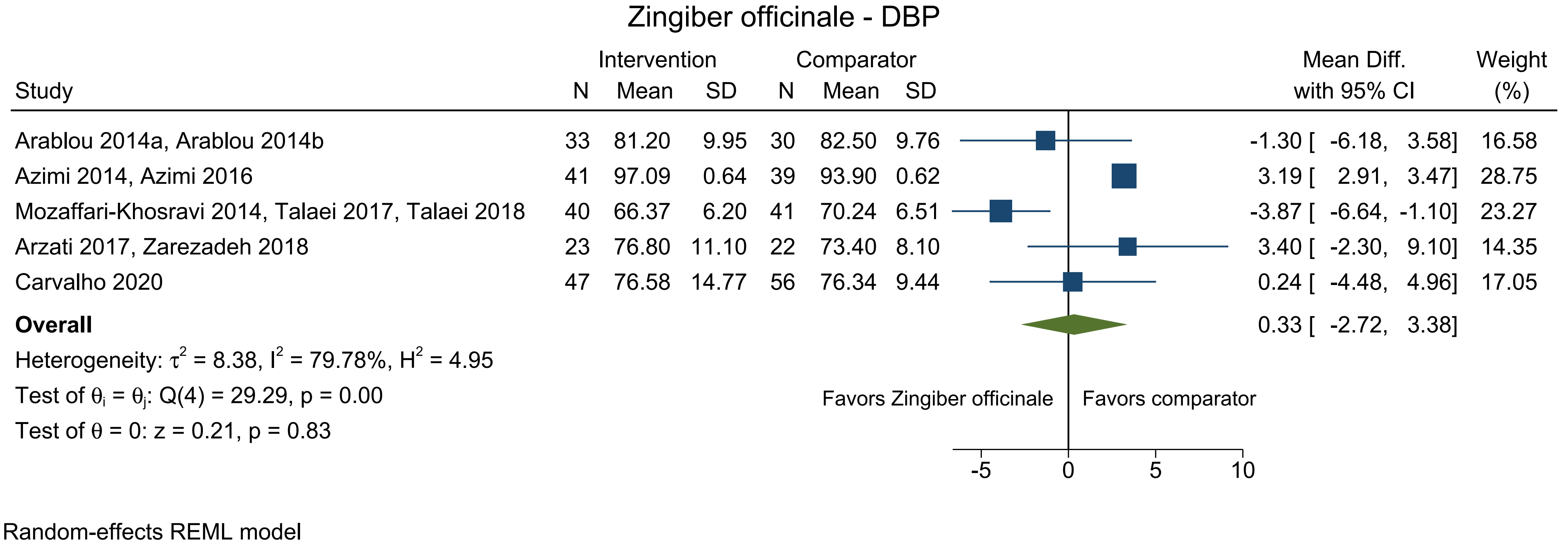

Zingiber officinale - DBP
Intervention
Comparator
Study
N
Mean
SD
N
Mean
SD
Mean Diff. with 95% CI
Weight (%)
Arablou 2014a, Arablou 2014b 33 81.20 9.95 30 82.50 9.76 -1.30 [-6.18, 3.58] 16.58
Azimi 2014, Azimi 2016 41 97.09 0.64 39 93.90 0.62 3.19 [2.91, 3.47] 28.75
Mozaffari-Khosravi 2014, Talaei 2017, Talaei 2018 40 66.37 6.20 41 70.24 6.51 -3.87 [-6.64, -1.10] 23.27
Arzati 2017, Zarezadeh 2018 23 76.80 11.10 22 73.40 8.10 3.40 [-2.30, 9.10] 14.35
Carvalho 2020 47 76.58 14.77 56 76.34 9.44 0.24 [-4.48, 4.96] 17.05
Overall 0.33 [-2.72, 3.38]
Heterogeneity: τ^2 = 8.38, I^2 = 79.78%, H^2 = 4.95
Test of $\theta_i = \theta_j$: Q(4) = 29.29, p = 0.00
Test of θ = 0: z = 0.21, p = 0.83
Favors Zingiber officinale
Favors comparator
-5
0
5
10
Random-effects REML model
